# Supplementary material for: Post-traumatic stress disorder and its associated factors among war-affected residents in Woldia town, North East Ethiopia, 2022; community based cross-sectional study
Source: PLoS One. 2023 Dec 21;18(12):e0292848. doi: 10.1371/journal.pone.0292848 (PMC10734942; doi:10.1371/journal.pone.0292848)
Supplement: Attachment — Submitted filename: Response to Reviewers.docx [file pone.0292848.s002.docx]

**Responses to the review’s comments**

**Dear PLOS ONE editorial team,**

Thank you for giving us the opportunity to submit a revised draft of the manuscript, and we would also like to thank you for your crucial comments on our paper **(**Manuscript ID: **PONE-D-22-34183**). We are very concerned and have combined all the suggested comments provided, which we believe strengthen our paper, and we hope this will render our paper eligible for consideration for publication in your reputed journal. We appreciate the time and effort that you and the reviewers dedicated to providing feedback on our manuscript and are grateful for the insightful comments and valuable improvements to our paper.

The author would like to inform you that we have addressed the comments and recommendations made by both reviewers and the editor, point by point. In addition, throughout our revision, we made our best corrections too. All changes in the revised manuscript are highlighted using tracking changes within the manuscript. Please see below, in blue, for a point-by-point response to the reviewers’ comments and concerns. All page numbers refer to the revised manuscript file with tracked changes.

**Comments from the editor:**

Dear Editor, Thanks for your timely and constructive comments and suggestions on our manuscript. We have incorporated the concerns and responded for your comments here below.

Journal Requirements:

When submitting your revision, we need you to address these additional requirements.

**#1…...**Please ensure that your manuscript meets PLOS ONE's style requirements, including those for file naming.

**Author response:** Thank you for your recommendations to adhere PLOS ONE’s style of requirements. We have ensured that the manuscript, its name, figures, and tables are per the journal requirements.

**#2…..**If you wish to make changes to your Data Availability statement, please describe these changes in your cover letter and we will update your Data Availability statement to reflect the information you provide.

**Author response:** Thank you for your suggestion and the dataset used and/or analyzed during the current study are available from the corresponding author on reasonable request.

Additional Editor Comments:

**#1…...**Please revise the references style according to the PLOS ONE submission guidelines.

**Author response:** Thank you very much for your comments. We did a revision on our referencing style and amend it based on the PLOS ONE submission guideline for referencing.

**#2…..**Ethics: Please provide information how the informed consent was retrieved from the participants

**Author response:** Thank you very much for your recommendation and we also noticed a lack of narration on the process of taking an informed consent. To make it clear, Data collection was done by trained data collectors by visiting each selected household and for those agreed they proceed to interview by putting their signature on the informed consent sheet and for few of those participants who were not literate, data collectors read the information sheet and explained to them when necessary, and assisted them to give finger print to confirm their consent. Additionally, the principal investigator of the study supervised the data collection process on daily base and performs a routine check on the questionnaires for consistency and completeness.

**#3……** The language needs to be improved and typos removed**.**

**Author response:** Thank you very much for your constrictive comment. We have tried with ultimate potential to improve the language usage and unnecessary typos were removed.

**#4……** In the introduction; summarize the current state of literature regarding the risk of specific exposures to traumatic events. Please use the existing literature to relate to your research questions, instead of listing the heterogeneous results on PTSD rates across different countries.

**Author response:** Thank you very much for your critical insight, and we shared your concern. We have revised the introduction by including exposure to a specific war-related traumatic events in different parts of the world that has a close relation with our research question.

**#5……** The co-occurrence of various trauma event types is high in war-affected area and should be reported and addressed in the results section.

**Author response:** Thank you and we appreciate your concern. We all know that in a war affected communities’ exposure to multiple war-related traumatic events was potentially high. We considered the co-occurrence of different trauma types as shown in (**page 11, Table 2)**. We categorize cumulative trauma exposure based on the number of exposures as, (1), (2-3) and (4 and above). We checked association in bi-variate logistic regression and found insignificantly associated with (p-value of 0.877) with the outcome PTSD. Another thing is, exposure to a single traumatic event is enough to be considered as exposed to war-related traumatic events.

Minor Comments

**#6……** Correct all concerning p-values to p <.001 (instead of p = 0.000)

**Author response:** Thank you very much for your suggestion, and we make correction on all concerning p-values to p <.001 (instead of p = 0.000).

**#7……** Evaluate all directions of comorbidity when discussion the high co-occurrence of disorders: Common Cause: Trauma predispose for depression and PTSD, PTSD results in Depression and Previous Depression renders the risk for individuals to develop PTSD.

**Author response:** Thank you very much for your clear insight. We tried to revise the high co-occurrence and interchanging association between exposure to traumatic events, PTSD and depression from different literatures.

**Response Reviewers’ comments**

**Reviewer #1**

Dear Reviewer #1, Thanks for your timely and constructive comments and suggestions on our manuscript. We have incorporated the concerns and responded for your comments here below.

**#1…...**In the study design and setting, it is better to cancel the sentence "The study aims to assess prevalence of post-traumatic stress disorder (PTSD) and its associated factors among war-affected residents in Woldia town".

**Author response:** Thank you very much for your constructive view. We shared your comment and the above stated sentence was removed from the study design and setting part.

**#2…...**You better to rewrite the exclusion criteria.

**Author response:** Thank you very much for the suggestion. We have modified the exclusion criteria in better and understandable way for the reader.

**#3….** Hence you have used the multistage sampling technique, why you did not consider the design effect while you calculate the sample size? Better to support the multistage in Figure(s).

**Author response:** we appreciate your critical view. yes, we use a multistage sampling technique and also consider the design effect, it was calculated and documented in the thesis documents but we noticed we didn’t include it in the submitted manuscript. So, to make it clear the sample size was determined by using a single population proportion by taking the prevalence of Post-traumatic stress disorder (PTSD) 59.8% from a study conducted in Northwest Ethiopia, following Maikadra Massacre(1) with; 5% margin of error, 95% confidence interval. Since, Multi-stage sampling technique was employed, by taking account design effect, the sample size was multiplied by 1.5. So, 369.4 x 1.5 = 554, adding 10% non-response rate total number samples becomes 609.

**#4…...** It is better if you tried to combine the strengths and limitations in one paragraph.

**Author response:** Thank you very much for your review and we have incorporated the strengths and limitations in one paragraph**.**

**#5…...** you better to acknowledge your participants rather than your co-authors.

**Author response:** Thank you very much for your clear question, and we take amendment and we express our deep-felt acknowledgment for our participants.

**Reviewer 2:**

Dear Reviewer #2, Thanks for your timely and constructive comments and suggestions on our manuscript. We have incorporated the concerns and responded for your comments here below.

**#1….** There is a general comment which is not addressed concerning the overall benefit of the manuscript. It is not clear why the authors choose especially that town because there is one existing article of another town in Northwest Ethiopia. It should be more clear why it is important to know the prevalence of another town and what the implications of these results are (e.g. regarding support services, practical implications).

**Author response:** Thank you very much for raising this issue. The rationale behind choosing Woldia town for PTSD assessment was the scope of armed conflict. During the armed conflict, Woldia town was highly affected demographically by the war, due to its strategic location for war, closeness to the border areas of Afar and Tigray regions, serious military surge and occupation for more than five months and several war related human right violations for relatively longer period of time than nearby towns. Therefore, Knowing the magnitude of PTSD and its predictors in woldia town will reveal sound and reliable picture to fill information gap. Moreover, this study used as input to identify affected groups, add clarity to the scope of war-related mental health burdens and helps the area through providing insight for stakeholders/voluntary organization to focus on post-war psychosocial rehabilitation for war affected woldia town residents.

**#2…...** In addition, the focus should also be more on PTSD subscales and subgroups (e.g. sex, educational status). Means and SD should be presented for PTSD and subscales and maybe also specific sociodemographic subgroups. At this point, the picture of the distribution of PTSD is too vague.

**Author response:** Thank you very much for your critical insight. For clarifying, our outcome of interest was PTSD with a binary outcome of having PTSD or not having PTSD. This is done based on the sum of 20 items from Post-traumatic stress disorder checklist (PCL-5) questioner with the cut-off point of 33. Since, the study uses a binary logistic regression results are explained in frequency and percent.

**#3…...** For publication, the manuscript needs a linguistic revision.

**Author response:** Thank you very much for your comment. As per your request, we have tried to revise the whole document for linguistic correction and we do with our best.

**Introduction**

**#1…...** In general, it is not quite clear why the authors choose especially this town – because there is at least one other publication from a city in Northwest Ethiopia. The introduction is lacking a narrative of why it is important to know this specific prevalence rate and what gap will be filled with these results. It would be great, if the authors could stress this in more detail.

**Author response:** Thank you very much for your concern. Actually this question is addressed in the above reviewer 2, **#**1.

**#2…...**The text says (line 64): “…are often part of the clinical picture of the illness”. Here, the authors stated the diagnostic criteria of PTSD regarding DSM and therefore, all the criteria mentioned must be part of the clinical picture of PTSD. This should be stated much clearer.

**Author response:** Thank you very much for your important view. On the revised introduction part (Page 3, line 2-7), we include most of characteristic symptoms under criteria B, C, D and E (based on DSM-5) in a clear and understandable way for the readers.

**#3…..**the authors name a lot of different studies, It would be better for the reader if the authors cite prevalence rates from meta-analyses or reviews, After stating these more global numbers, it would be perfect to name single prevalence studies from either region similar to the situation in Woldia or others from Ethiopia. That depends on the aim of the authors. It might also be a benefit if the authors focus only on adults in their literature

**Author response:** we really thank you and appreciate your concern. Based on your comment we put related literatures like global figures, meta-analysis, national and regional survey and pooled prevalence in a single cluster. Then we describe the prevalence of PTSD across different countries of the world from the farthermost to the closest based on geographical location.

**#4….** for the reader it would be easier to cluster all these factors in sociodemographic, trauma-related and psychopathological factors.

**Author response:** we are grateful for your comment. Based on your suggestion we cluster factors in Sociodemographic, medical and clinical factors, as shown in (page 5, line 4-7).

**#5…..** How did the authors assess and decide if a person is to severely sick or unable to communicate?

**Author response:** Thanks for pointing out for clarification. Since the data were gathered through face-to-face interview, individuals who/ family member/care givers reported that they were severely ill to give an interview for data collectors, those with medical certificate or related document that brief the current illness of the patient and those with observable severe physical illness/verbal communication problem were decided as not eligible for the study.

**#6….** Could you please insert the Figure 1 in the text to have a reference in the text for the figure? That would be very helpful.

**Author response:** Thank you for your recommendation. As per the guideline of PLOS ONE journal Figures should be uploaded separately as individual file. But, based on your comment we insert the caption of Figure 1 shown in (page 6, line 4-6).

**#7….**  it would be better to set the citation for the questionnaire directly after the first mention, i.e. (PLC-5, “citation of author of the questionnaire”).

**Author response:** We appreciate your comment. As you recommend we put the citation of author of the questionnaire directly after the first mention, as shown in (page 7, line 17).

**#8…** the same for the HTQ. It would be easier to identify the source if it directly follows naming the questionnaire (HTQ, "source"). The same for Line 175, Line 179, line 184.

**Author response:** we acknowledge your comment and we amend all of the above listed line numbers as your comment. As shown in (page 7, line 25, line 28) and (page 8, line 3) respectively.

**#9….** Is the questionnaire (Oslo 3-item social support scale) originally quoted in the mentioned literature (34)?

**Author response:** we really appreciate your in depth view and we are shocked because it was not even cited in the original document (final thesis) and definitely it was an error. With apology, the comment is accepted and the cite was corrected with the right reference.

**#10….** the authors stated that “sociodemographic, substance use history and clinical factors were operationalized according to different literature”. The (35) literature refers primarily to alcohol and drug abuse. Is this source also referring to other clinical factors?

**Author response:** Thank you for your suggestion. Actually literature (35) refers only for alcohol and drug abuse and we missed citing sociodemographic and clinical factors. As you suggest we put citation for those factors, as shown in (page 8, line 8).

**#11….** I do not fully understand why you first checked variables in a bivariate logistic regression and then enter them in a multivariate logistic regression? What is the rationale behind this? The second question refers to the p-value of <0.2. Why do you use this p-value?

**Author response:** We appreciated your comment. Our study has binary outcome (PTSD/ not PTSD) and uses a binary logistic regression model for the analysis. So, we identify participants with a minimum of exposure to one traumatic event by using Harvard Trauma Exposure(HTE) tool. For the analysis Forward selection method was used and in the first stage we checked the association between each variable with the outcome (PTSD) to identify significant predictors of PTSD and for those variables with p-value <0.2 (if adequate factors associated) or in some cases <0.25 (if few factors only associated) they will taken to multivariate logistic regression for further analysis to control possible confounding variables. Those variables with a p-value <0.05 considers as significantly associated and their association will be explained by AOR (Adjusted odds ratio).

**Result**

**#12….** In general, the authors could decide which information they want to state in the text. At the moment, every result in the text has its counterpart in a table. Maybe there is some space to shorten the text.

**Author response:** Thank you for your comment. we have narrated in shortened way.

**#13….** a minor comment: please add “years” in the column for “Age”.

**Author response:** Thank you for your comment. we add “years” in the column for “Age”.

**#14….**It is very interesting that 35.34% of the participants are government employees as well as that 55% have children. are these typical numbers for Woldia? It would be great if you can say something in the discussion.

**Author response:** Thank you for your important view. Those results are typical numbers we found through the study and I feel your opinion on these two factors, being government employee and having children. Actually, having children was purposely assessed factor after we found its significant association with PTSD in various literatures. During analysis, we checked its association in bi-variate logistic regression and we found p-value of 0.668, which not associated with the outcome of interest and rejected from further analysis. The same is true for occupation (government employee), while checked we found p-value of 0.249, so it was rejected from further analysis. Due to this, we couldn’t say anything on the result or discussion part.

**#15….**Table 2: it is not quite clear why the authors split the variable “way of exposure” into male and female but not the variable “exposure to TE”.

**Author response:** Thank you for your critical view. yes, we agree that, we don’t have a clear reason or intention for not classifying ways trauma exposure among male and females. Based on your comment we show distribution of trauma exposure among male and females. Here, we want to explain that, we use the Harvard Trauma Exposure(HTE) questioner for two main purposes, one is for identifying participants for exposure to war-related traumatic events and second is for which specific type of traumatic event he/she is exposed, then we assess the symptoms of PTSD by using PCL-5 questioner and check the significance of association with the outcome of interest (PTSD).

**#16….** It would be great to also have the mean scores (and SD) for depression, anxiety and social support.

**Author response:** We thank you for your recommendation. We use a standardized tool with a known cut-off point, as shown in (page 7, line 25), (page 8, line 3 and 8). We use Patient Health Questionnaire item nine (PHQ-9) for assessing depression with a cut-off point of 10, generalized anxiety disorder (GAD-7) seven item tool with a cut-off point of 10 and oslo- 3 social support scale with a scoring of 3–8=poor social support; 9–11=moderate social support; and 12–14=strong social support. Due to this, putting the mean score and SD for the above tools was not relevant.

**#17….** It is important to also have the mean score (and SD) for PTSD, the means for the clusters and maybe also all these means with regard to different sociodemographic variables (e.g. sex: male/female, education).

**Author response:** We appreciate for pointing out this important view. PCL-5 was a 20 item tool, measures the 20 DSM-5 symptoms of Post-Traumatic Stress Disorder. A total score is computed by adding the 20 items, so that possible scores range from 0 to 80 with a five Likert scale (0= Not at all, 1= A little bit, 2 =Moderately, 3=Quite a bit, 4= Extremely) with a cut-off point of ≥33. Those scored below 33 will be considered not having PTSD and those with 33 and above was taken as having PTSD. Since, the tool has its own cut-off point it is not significant to mention the mean score and SD.

**#18….**  I have to ask under which assumptions only these named variables fulfil minimum requirements for further analysis. In addition, the p value less than 0.2 should also be explained.

**Author response:** Thanks for your comment and this question is addressed in **#11.**

**#19….** With regard to the different traumatic events, I am curious why sexual assault seems to be not relevant in this context. Do you have an explanation?

**Author response:** Thanks for pointing out for clarification. We strongly believe on the relevance of assessing sexual assault/rape as a contributing factor, but before conducting the actual data collection we done a pre-test by taking 5% of the sample size (31 questioners) in Dessie town and we noticed such private and sensitive questions related to sexual abuse/assault and rape has raised social desirability issue, under-reporting and participants are not even comfortable to proceed with the rest of the question. Due to this we are afraid that this may affect the truthfulness of other information and we decide to remove but we mentioned this in the limitation part (page 18, line 14-18) and we recommend interested researchers to fill this gap.

**#20….** Since the individual categories being compared here are not exclusive (i.e., a person who experienced x may have experienced y as well), These persons can be completely overlapping. Was this considered in the calculation?

**Author response:** Thank you very much for raising this issue. Its known that, in war and conflict situation exposure to a multiple traumatic event is potentially high, due to this we assesse a cumulative trauma (frequency of traumatic event) and described in (page 11, Table 2) and its considered in the calculation. In addition, PCL-5 questioner has an option that requests participants to focus one and worst event among all traumatic events before proceeding to asking symptoms, so participants might be exposed to different types of war-related traumatic events but, expected to think and rehearse on one event and tool asses PTSD, depression and anxiety symptoms based on their experience.

**#21….** It does not have to be so detailed when it comes to the classification of the prevalence rate. I think it would be sufficient to state 1-2 sources with similar background and state that there are also other rates with a similar population but that different reasons have an influence on the rates.

**Author response:** Thank you very much for your constructive comment. We have amended the discussion part based on your recommendation.

**#22….** I am not sure if the authors want to refer to acute stress as a response to destruction/looting or to PTSD.

**Author response:** Thank you very much for pointing out this issue. We understood the explanation in this way, for those individuals with a destruction/looting of personal property creates psychological distress by itself and a significant or difficulty to replace worse the symptoms. A recurrent rehearsal and rumination of the traumatic event becomes their daily experience and some individuals deal effectively but others become impaired and exposed to other mental illnesses including acute stress disorder. In long term untreated cased leads to be post-traumatic stress disorder.

**#23…..** did the authors ask about the examples of the intrusive thoughts or are these assumptions they made due to the experience of traumatic events?

**Author response:** Thank you very much for your concern. During the data collection, there were challenging situations specially while facing those who lost their close family members/friends. Most of them talks with disturbed emotion, tearful, sorrow, guilty feelings and anger. We understand the form their words, emotion, facial expression and thoughts that most of them have thought of revenge, anger and self-blame. Additionally, we assume in such un-natural death of family members/friend negative intrusive thought and feeling of revenge might be there, so with both their reporting and our assumption intrusive thoughts may be experienced.

**#24….** a source is needed for the stated fact.

**Author response:** Thanks for your critical view. we put a reference for the stated fact.

**#25….** There are other limitations to this study, e.g. that PTSD

**Author response:** Thank you very much for your comment. Based on your comment we revised the literature and add additional imitations. As shown in (page 18, line 10-18).

1.	Ali D, Azale T, Wondie M, Tadesse JJPr, management b. About six in ten survivors of the november 2020 Maikadra massacre suffer from posttraumatic stress disorder, northwest Ethiopia. 2022;15:251.
