# Supplementary material for: Post-traumatic stress disorder and its associated factors among war-affected residents in Woldia town, North East Ethiopia, 2022; community based cross-sectional study
Source: PLoS One. 2023 Dec 21;18(12):e0292848. doi: 10.1371/journal.pone.0292848 (PMC10734942; doi:10.1371/journal.pone.0292848)
Supplement: Attachment — Submitted filename: Response to Reviewers.docx [file pone.0292848.s003.docx]

**Responses to the review’s comments- Second Round**

**Dear PLOS ONE editorial team,**

We would like to appreciate for your quick response and constructive comments on our paper **(**Manuscript ID: **PONE-D-22-34183**). We are very concerned for all the raised comments, which we believe strengthen our paper, and we hope this will render our paper eligible for consideration for publication in your reputed journal. We appreciate the time and effort that you and the reviewers for providing feedback on our manuscript and we are grateful for the insightful comments and valuable improvements to our paper.

I, the author would like to inform you that, we have fully accepted the comments and made amendments based on your recommendations made by the Academic editor and both reviewers. In addition, throughout our revision, we made a point-by-point corrections with our level best too. All changes in the revised manuscript are highlighted using tracking changes within the manuscript. Please see below, in blue, for a point-by-point response to the Academic editor and reviewers’ comments and concerns. All page numbers refer to the revised manuscript file with tracked changes.

**Comments from the editor:**

Dear Editor, Thanks for your constructive comments and suggestions on our manuscript. We have incorporated the concerns and responded for your comments here below.

Journal Requirements:

Addressing journal requirements.

**#1…...**Please ensure consistency in capitalization throughout the manuscript, proper use of tittle case for headings and subheadings, and lower case for regular text.

**Author response:** Thank you for your suggestion to adhere PLOS ONE’s requirements. We have reviewed and ensure that the manuscript uses a consistent capitalization, tittle case for heading and subheadings and lower case for regular text.

**#2…..**Please review your reference list to ensure that it is complete and correct.

**Author response:** Thank you for your comment and we have checked our reference list repeatedly for its correctness and completeness but we couldn’t found incomplete or retracted references.

Additional Editor Comments:

**#1…...**Please be clear about your cumulative measure of cumulative trauma, rational for its categorization and correlation with the outcome PTSD.

**Author response:** Thank you very much for your critical comments. Cumulative trauma was added after a careful revision from different studies. At the beginning, we believe and found evidence of the dose-response association of multiple exposure to traumatic event with PTSD from various studies and decided to assess it in our study by taking the range of categorization from familiar studies conducted in Ethiopia. During analysis stage we checked its correlation in bivariate logistic regression and found a (p-value of 0.877) and rejected from further analysis. Additionally, score of cumulative trauma was defined with its rational in the method section (page 7, line 10-13) and the result was highlighted in trauma exposure section (page 9, line 5-7).

**#2…..**Please clarify the ”way of exposure”. Is this type of exposure related to the worst event?

**Author response:** Thank you very much for raising, based on DSM-5, “way of exposure” is component of criteria “A” of the diagnostic criteria. Way of exposure can happen in four means, some individuals directly experience the event on themselves (e.g. Physical trauma/ Serious injury), others might witness the event in person (e.g. Witnessing the murder), by learning that the event occurred to a close family/friend was the other means of exposure and few was exposed as part of their job like police officers, nurses and fire responders. So way of exposure is a means or situation through which individuals get exposed to the event. Multiple studies support the direct experience of the event increases the chance of having PTSD as compared with other means of exposure and we assesse” way of exposure” both as part of diagnostic criteria and assessing its correlation with PTSD. During the interview, we assesse individuals for exposure by using Harvard trauma questioner (HTQ) and while encountered with individuals exposed for multiple events we recommend to think of the worst one event then we proceed with the questions.

**#3……** Consider another studies about the importance of sexual violence in North East Ethiopia**.**

**Author response:** Thank you very much for your constrictive comment.

**#4**……. Please use references for the fact of social desirability and underreporting of sexual violence. It may be useful to encourage future studies to assess exposure to sexual violence.

**Author response:** Thank you for concern, social desirability and underreporting of sexual violence items was experienced during our pretest study before the actual data collection, so to encourage researchers we put it as a drawback of our study and we strongly recommend other studies to include rape and sexual violence as part of traumatic events and since such questions are sensitive and private we recommend to employ qualitative study design and sampling procedures e.g. Snowball sampling techniques.

**#5……** Clarify why you have put some numbers in bold. (In table 3)

**Author response:** Thank you very much for your critical view, we don’t have any intention rather than indicating variables that significantly associated with the outcome variable. To adhere with the PLOS ONE’s requirements we un bold those numbers.

**#6……** Comments on proper use of upper and lower cases.

**Author response:** Thank you and we appreciate your concern. We have made a review and as much as possible we done amendments and use proper cases.

**Review comments to the author**

**Response to Reviewers’ comments, second round.**

**Reviewer #1**

Dear Reviewer #1, Thanks for your comments and suggestions on our manuscript. We have incorporated the concerns and responded for your comments here below.

**#1…...**I recommend to the authors to send a point by point response for each questions that has been raised in the previous round.

**Author response:** Thank you very much for your comment. In the previous round, we have done a point-by-point response for each questions and submitted the revised version of the manuscript based on the allotted time and if it’s necessary we can resend the “Response to Reviewers” document. Thanks again for the comment.

**Reviewer 2:**

Dear Reviewer #2, Thanks for your timely and constructive comments and suggestions on our manuscript. We have incorporated the concerns and responded for your comments here below.

**#1….** In the Introduction section, you only used some parts of the explanation, but I find it important to include the whole explanation as you send it.

**Author response:** Thank you for constructive comment. We have tried to explain the nature of conflict, importance of our study and its significance for respective stakeholders.

**#2…...** Thank you for your explanation regarding the binary outcome of PTSD.

**Author response:** Thank you very much for your comment.

**#2…...** Could you additionally include that you refer to the DSM-5 PTSD criteria? That would be helpful to know it right away without looking at the reference. Thank you.

**Author response:** Thank you very much for your comment. In the introduction, we use the standardized definition of PTSD sourced from DSM-5 and cited appropriately. Based on your comment, we mention our reference (DSM-5) for defining PTSD.

**#3…...** Thank you for including global figures and other surveys. This improved the introduction. For a more focused narrative, I would suggest deleting the studies from Ukraine, Syria and Lebanon because you refer to so many other countries in Africa that this already makes your point.

**Author response:** Thank you very much for your suggestion. To show the global burden, we have tried to highlight studies conducted from different corners of the world and we gave a strong emphasis on studies conducted in Africa.

**#4…...** In addition, I would like to encourage you to add more details in the section where you address contributing factors to the occurrence of PTSD (ll 121-128) if they are from studies conducted in Africa

**Author response:** Thank you very much for your important view. most studies referenced in the contributing factors are studies conducted in Africa including Ethiopia as shown (Page 4, line 13-19).

**#5…..** Thank you for explaining the criteria regarding “severely sick” or “unable to communicate”. It would be helpful if you include this explanation also in the manuscript.

**Author response:** We really thank you and appreciate. Based on your suggestion, we took amendments on the exclusion criteria.

#11 and #18 Thank you for the clarification.

**Author response:** we are grateful for your comment.

Additional new comments:

Table 2: please add the frequencies for male and female in the columns. At the moment you report the sample size for male and female but due to different sizes, it is hard to compare both columns. You already have the frequency for the total sample and it would be helpful to also have it for male/female.

**Author response:** Thanks for pointing out and as shown in (Table 2), we have described the frequency based on sex (male/female) for each result in the columns.

Table 3: please include the abbreviation AOR and CI in the notes below the table.

**Author response:** Thank you for your recommendation. We have included the all abbreviations used in the manuscript in the List of Abbreviation section, as shown in (page 18, line 5-8).
