# Supplementary material for: Post-traumatic stress disorder and its associated factors among war-affected residents in Woldia town, North East Ethiopia, 2022; community based cross-sectional study
Source: PLoS One. 2023 Dec 21;18(12):e0292848. doi: 10.1371/journal.pone.0292848 (PMC10734942; doi:10.1371/journal.pone.0292848)
Supplement: Attachment — Submitted filename: Response to Reviewers.docx [file pone.0292848.s004.docx]

**Full and Complete Revision of Comments Raised by the Academic Editor and Reviewers**

**Dear PLOS ONE editorial team,**

We would like to express our gratitude for the chances you give in order to submit fully and completely revised version of the manuscript **(**Manuscript ID: **PONE-D-22-34183**). We are committed to submit a fully revised manuscript by compiling and responding to all of the comments raised in the past two rounds by the academic editor and both reviewers.

We strongly believe that all the raised comments and suggestions provided will strengthen and enhance the quality of our paper, and we hope this will render our paper eligible for publication in your reputed journal. We appreciate the time and effort that you and the reviewers providing insightful comments and feedbacks on our manuscript and are grateful for valuable improvements to our paper.

We author would like to state that we have made a full revision of all of comments point by point raised in the previous two rounds and to make responses clear we incorporate all of comments by classifying them in sections, as “Section One” for comments raised in the first round and “Section Two” for the comments raised in the second round. We also incorporate comments raised by Academic editor in the last e-mail. We emphasize that, throughout our revision, we made a careful amendment and addressed the comments and suggestions point by point raised by both reviewers and academic editor.

All changes in the revised manuscript are highlighted using tracking changes within the manuscript. Please see below, in blue, for a point-by-point response to the reviewers’ comments and concerns. All page and line numbers refer to the revised manuscript with tracked change file.

# Section One (Response to comments raised in the first round)

## **Comments from the editor:**

Dear Academic Editor, Thank you for your constructive comments and suggestions on our manuscript. We acknowledged all your concerns and we responded for your comments here below.

Journal Requirements:

**#1** Please ensure that your manuscript meets PLOS ONE's style requirements, including those for file naming.

**Author response:** Thank you for your recommendations, to adhere PLOS ONE’s style of requirements. We have ensured that the manuscript, its name, figures, and tables are as per the journal requirements.

**#2** If you wish to make changes to your Data Availability statement, please describe these changes in your cover letter and we will update your Data Availability statement to reflect the information you provide.

**Author response:** Thank you for your suggestion and we have stated in the data availability section as “The dataset used and/or analyzed during the current study are submitted to the journal as supplementary information and can be found, in the online version, at doi.org/10.21203/rs.3.rs-2319793/v1.”

**#3** Please revise the references style according to the PLOS ONE submission guidelines.

**Author response:** Thank you for your comment. We did a revision on our referencing style and amend it based on the PLOS ONE submission guideline.

**#4** Ethics: Please provide information how the informed consent was retrieved from the participants

**Author response:** Thank you very much for your concern and we also noticed a lack of narration on the process of taking an informed consent. To make it clear, Data collection was done by trained data collectors by visiting each selected household and for those agreed they proceed to interview by putting their signature on the informed consent sheet and for few of those participants who were not literate, data collectors read the information sheet and explained to them when necessary, and assisted them to give finger print to confirm their consent. Additionally, the principal investigator of the study supervised the data collection process on daily base and performs a routine check on the questionnaires for consistency and completeness.

**#5** The language needs to be improved and typos removed**.**

**Author response:** Thank you very much for your constrictive comment. We made frequent revision to improve language usage and to remove unnecessary typos.

**#6** In the introduction; summarize the current state of literature regarding the risk of specific exposures to traumatic events. Please use the existing literature to relate to your research questions, instead of listing the heterogeneous results on PTSD rates across different countries.

**Author response:** Thank you very much for your critical insight. We revise the introduction by stating exposure to a specific war-related traumatic events in different parts of the world that has a close relation with our research question.

**#7** The co-occurrence of various trauma event types is high in war-affected area and should be reported and addressed in the results section.

**Author response:** Thank you and we appreciate your concern. We agree that in a war affected communities’ exposure to multiple war-related traumatic events was potentially high. For this purpose, we assesse cumulative trauma, (page 8, Line 211-214) exposure to the number of different types of traumatic events as, (1), (2-3) and (4 and above) traumatic events. In result section, we report regarding cumulative traumas as shown (page 10, Line 266-268**)** and (page 11, Table 2**)**.

**#8** Correct all concerning p-values to p <.001 (instead of p = 0.000)

**Author response:** Thanks for your suggestion, and we make correction on all concerning p-values to p <.001 (instead of p = 0.000).

**#9** Evaluate all directions of comorbidity when discussion the high co-occurrence of disorders: Common Cause: Trauma predispose for depression and PTSD, PTSD results in Depression and Previous Depression renders the risk for individuals to develop PTSD.

**Author response:** Thank you for your clear insight. We reviewed the high co-occurrence and interchanging association between exposure to traumatic events, PTSD and depression from different literatures and we incorporate it in the discussion section (page 17, Line 374-375) and (page 17, Line 380-383).

## Reviewers’ comments to the Author

**Reviewer # 1:**

Dear Reviewer #1, Thanks for your constructive comments and suggestions on our manuscript. We have incorporated all concerns and addressed for your comments here below:

**#1** In the study design and setting, it is better to cancel the sentence "The study aims to assess prevalence of post-traumatic stress disorder (PTSD) and its associated factors among war-affected residents in Woldia town".

**Author response:** Thank you for your constructive view. based on your suggestion the above stated sentence was removed from the study design and setting part. (page 6, Line 150-151).

**#2** You better to rewrite the exclusion criteria.

**Author response:** Thank you for the suggestion. We have modified the exclusion criteria in better and understandable way for the reader, as shown (page 6, Line 164-167).

**#3** Hence you have used the multistage sampling technique, why you did not consider the design effect while you calculate the sample size? Better to support the multistage in Figure(s).

**Author response:** we appreciate your critical view. Yes, we use a multistage sampling technique and also consider the design effect, it was calculated and documented in the thesis documents but we noticed we didn’t include it in the submitted manuscript. So, to make it clear the sample size was determined by using a single population proportion by taking the prevalence of post-traumatic stress disorder (PTSD) 59.8% from a study conducted in Northwest Ethiopia, following Maikadra Massacre[1] with; 5% margin of error, 95% confidence interval. Since, Multi-stage sampling technique was employed, by taking account design effect, the sample size was multiplied by 1.5. So, 369.4 x 1.5 = 554, adding 10% non-response rate total number samples becomes 609. To show the sampling procedure, we use (Fig 1). A schematic representation and proportional allocation of samples to the randomly selected administrative units(kebeles) as shown (page 6-7, Line 157-183).

**#4** It is better if you tried to combine the strengths and limitations in one paragraph.

**Author response:** Thank you very much for your review and as per your comment we have incorporated the strengths and limitations of the study in one paragraph**.** (page 18, Line 415-424)

**#5** You better to acknowledge your participants rather than your co-authors.

**Author response:** Thank you for your clear question, and we take amendment and we express our deep-felt acknowledgment for our participants. (page 19, Line 430-431)

**Reviewer # 2:**

Dear Reviewer #2, Thanks for your timely and constructive comments and suggestions on our manuscript. We have incorporated all concerns and responded for your comments here below:

**#1** There is a general comment which is not addressed concerning the overall benefit of the manuscript. It is not clear why the authors choose especially that town because there is one existing article of another town in Northwest Ethiopia. It should be more clear why it is important to know the prevalence of another town and what the implications of these results are (e.g. regarding support services, practical implications).

**Author response:** Thank you very much for raising. The rationale behind choosing Woldia town for PTSD assessment was the scope of armed conflict. During the armed conflict, Woldia town was highly affected demographically by the war, due to its strategic location for war, closeness to the border areas of Afar and Tigray regions, serious military surge and a five-month encroachment and several war related human right violations for relatively longer period of time than nearby towns. Therefore, knowing the magnitude of PTSD and its predictors in woldia town will reveal sound and reliable picture to fill information gap. Moreover, this study used as input to identify affected groups, add clarity to the scope of war-related mental health burdens and helps the area through providing insight for stakeholders/voluntary organization to focus on post-war psychosocial rehabilitation for war affected woldia town residents.

**#2** In addition, the focus should also be more on PTSD subscales and subgroups (e.g. sex, educational status). Means and SD should be presented for PTSD and subscales and maybe also specific sociodemographic subgroups. At this point, the picture of the distribution of PTSD is too vague.

**Author response:** Thank you for your critical insight. For clarifying, our outcome PTSD has only a binary outcome, weather of having PTSD or not having PTSD. This is calculated based on the sum of 20 items scored out of 80 from post-traumatic stress disorder checklist (PCL-5) questioner with the cut-off point of 33. Individuals who score 33 and above are considered as having PTSD and vice versa. Due to this, we didn’t put the Means score and SD of PTSD. Additionally, the tool has no severity scale to show how much of them has mild, moderate or severe level of the illness.

**#3** For publication, the manuscript needs a linguistic revision.

**Author response:** Thank you very much for your comment. As per your request, we have done frequent revision to the whole document for linguistic correction.

**#4** In general, it is not quite clear why the authors choose especially this town – because there is at least one other publication from a city in Northwest Ethiopia. The introduction is lacking a narrative of why it is important to know this specific prevalence rate and what gap will be filled with these results. It would be great, if the authors could stress this in more detail.

**Author response:** Thank you very much for your concern. Actually this question is addressed in the above question **#**1.

**#5** The text says (line 64): “…are often part of the clinical picture of the illness”. Here, the authors stated the diagnostic criteria of PTSD regarding DSM and therefore, all the criteria mentioned must be part of the clinical picture of PTSD. This should be stated much clearer.

**Author response:** Thank you very much for your important view. On the revised introduction part (Page 3, Line 62-68), we include most of characteristic symptoms under criteria B, C, D and E (based on DSM-5) in a clear and understandable way for the readers.

**#6** Line 81-112: The authors name a lot of different studies, it would be better for the reader if the authors cite prevalence rates from meta-analyses or reviews, after stating these more global numbers, it would be perfect to name single prevalence studies from either region similar to the situation in Woldia or others from Ethiopia. That depends on the aim of the authors. It might also be a benefit if the authors focus only on adults in their literature

**Author response:** we appreciate your concern. Based on your comment we put related literatures like global figures in one cluster. Then we describe the prevalence of PTSD across different countries of the world from the farthermost to the closest based on geographical location and give emphasis on studies conducted in Africa in similarity with the situation in Woldia town and we focus on studies done on adults.

**#7** Line 113-119: For the reader it would be easier to cluster all these factors in sociodemographic, trauma-related and psychopathological factors.

**Author response:** we are grateful for your comment. Based on your suggestion we cluster factors in Sociodemographic, clinical and trauma related factors as shown in (page 5, Line 122-129).

**#8** Line 141/142: How did the authors assess and decide if a person is too severely sick or unable to communicate?

**Author response:** Thanks for pointing out for clarification. Since the data were gathered through face-to-face interview, individuals who/ family member/care givers reported that they were severely ill to give an interview for data collectors, those with medical certificate or related document that brief the current illness of the patient and those with observable severe physical illness/verbal communication problem were decided as not eligible for the study.

**#9** Could you please insert the Figure 1 in the text to have a reference in the text for the figure? That would be very helpful.

**Author response:** Thank you for your recommendation. As per the guideline of PLOS ONE journal Figures should be uploaded separately as individual file. But, based on your comment we insert the caption of Figure 1 shown in (page 6, Line 157-159).

**#10** Line 164: it would be better to set the citation for the questionnaire directly after the first mention, i.e. (PLC-5, “citation of author of the questionnaire”).

**Author response:** We appreciate your comment. As you recommend we put the citation of author of the questionnaire directly after the first mention, as shown in (page 7, Line 200).

**#11** Line 172: the same for the HTQ. It would be easier to identify the source if it directly follows naming the questionnaire (HTQ, "source"). The same for Line 175, Line 179, line 184.

**Author response:** We acknowledge your comment and we amend all of the above listed line numbers as your comment. As shown in (page 8, Line 208, 215 and 219) respectively.

**#12** Line 184: Is the questionnaire (Oslo 3-item social support scale) originally quoted in the mentioned literature (34)?

**Author response:** we really appreciate your in depth view and we are shocked because it was not even cited in the original document and definitely it was an error. With apology, the comment is accepted and the cite was corrected with the right reference as shown (page 8, Line 223).

**#13** Line 187-188: The authors stated that “sociodemographic, substance use history and clinical factors were operationalized according to different literature”. The (35) literature refers primarily to alcohol and drug abuse. Is this source also referring to other clinical factors?

**Author response:** Thank you for your suggestion. Actually literature (35) refers only for alcohol and drug abuse and we missed citing sociodemographic and clinical factors. As you suggest we put citation for those factors, as shown in (page 8, Line 227).

**#14** Line 191-193: I do not fully understand why you first checked variables in a bivariate logistic regression and then enter them in a multivariate logistic regression? What is the rationale behind this? The second question refers to the p-value of <0.2. Why do you use this p-value?

**Author response:** We appreciated your comment. Our study has binary outcome (PTSD/ not PTSD) and uses a binary logistic regression model for the analysis. For the analysis Forward selection method was used and in the first stage we checked the association between each variable with the outcome (PTSD) to identify significant predictors of PTSD and for those variables with p-value <0.2 (if adequate factors associated) or in some cases <0.25 (if few factors only associated) they will take to multivariate logistic regression for further analysis to control possible confounding variables and minimize statistical error. Those variables with a p-value <0.05 considers as significantly associated and their association will be explained by AOR (Adjusted odds ratio).

**#15** In general, the authors could decide which information they want to state in the text. At the moment, every result in the text has its counterpart in a table. Maybe there is some space to shorten the text.

**Author response:** Thank you for your comment. we have narrated the result in shortened way.

**#16** Table 1: A minor comment: please add “years” in the column for “Age”.

**Author response:** Thank you for your comment. we add “years” in the column for “Age”.

**#17** It is very interesting that 35.34% of the participants are government employees as well as that 55% have children. are these typical numbers for Woldia? It would be great if you can say something in the discussion.

**Author response:** Thank you for your important view. Those results are typical numbers we found through the study and I feel your opinion on these two factors, being government employee and having children. Actually, having children was purposely assessed factor after we found its significant association with PTSD in various literatures. During analysis, we checked its association in bi-variate logistic regression and we found p-value of 0.668 and rejected from further analysis. The same is true for occupation (government employee), while checked we found p-value of 0.249. Since those factors doesn’t associate we didn’t see its significance of discussing.

**#18 Table 2:** it is not quite clear why the authors split the variable “way of exposure” into male and female but not the variable “exposure to TE”.

**Author response:** Thank you for your critical view. We don’t have a clear reason or intention for not classifying exposure to traumatic event in to male and females. So, we add distribution of exposure to traumatic event among both sex in the column. As shown (page 10-11, Table 2).

**#19** In general, for a better understanding it would be helpful to state that the “way of exposure” refers to the most traumatic event but the variable “exposure to traumatic event” is not.

**Author response:** Thank you for your clarification. To show the difference, “way of exposure” was one of the criteria for the diagnosis of PTSD based on DSM-5. It is the means through which the individual is exposed to the feared situation. Way of exposure could happen in different means, experiencing the event on themselves, through direct witnessing, by learning or exposed as part of their job. “exposure to traumatic event” is simply for which traumatic event is the person exposed for. HTQ tool list nine commonly reported traumatic events and we assesse for which one is the participant exposed, it can be single or multiple traumatic event and we request to think of the worst one and proceed to the interview.

**#20** It is not clear why the authors only listen certain traumatic events but not all. It would be helpful for the reader to have an overview of all traumatic events (it could be in the supplement section).

**Author response:** Thank you for your concern. For assessment of war related traumatic event, we use Harvard trauma questioner (HTQ), used in different countries including Ethiopia. It contains nine commonly reported traumatic events and individuals might have exposed for one, two or above events.

**#21** Line 225-231: It would be great to also have the mean scores (and SD) for depression, anxiety and social support.

**Author response:** We thank you for your recommendation. We use a standardized tool with a known cut-off point, as shown in (page 7, Line 215-225). We use Patient Health Questionnaire item nine (PHQ-9)[2] for assessing depression with a cut-off point of 10, generalized anxiety disorder (GAD-7)[3] seven item tool with a cut-off point of 10 and Oslo- 3 social support scale[4] with a scoring of 3–8=poor social support; 9–11=moderate social support; and 12–14=strong social support. Due to this, putting the mean score and SD for the above tools was not relevant.

**#22** Line 233-235: It is important to also have the mean score (and SD) for PTSD, the means for the clusters and maybe also all these means with regard to different sociodemographic variables (e.g. sex: male/female, education).

**Author response:** We appreciate for pointing out this important view. PCL-5[5] was a 20 item tool, measures the 20 DSM-5 symptoms of post-traumatic stress disorder. A total score is computed by adding the 20 items, so that possible scores range from 0 to 80 with a five Likert scale (0= Not at all, 1= A little bit, 2 =Moderately, 3=Quite a bit, 4= Extremely) with a cut-off point of ≥33. Those scored below 33 will be considered not having PTSD and those with 33 and above was taken as having PTSD. Since, the tool has its own cut-off point it is not significant to mention the Mean score and SD for PTSD.

**#23** Line 236-241: I have to ask under which assumptions only these named variables fulfil minimum requirements for further analysis. In addition, the p value less than 0.2 should also be explained.

**Author response:** Thanks for your comment and this question is addressed in **#14.**

**#24** With regard to the different traumatic events, I am curious why sexual assault seems to be not relevant in this context. Do you have an explanation?

**Author response:** Thanks for pointing out for clarification. We strongly believe on the relevance of assessing sexual assault/rape as a contributing factor, but before conducting the actual data collection we done a pre-test by taking 5% of the sample size (31 questioners) in Dessie town and we noticed such private and sensitive questions related to sexual abuse/assault and rape has raised social desirability issue, under-reporting and participants are not comfortable to proceed with the rest of the question. Due to this we are afraid that this may affect the truthfulness of other information and we decide to remove but we mentioned this in the limitation part (page 18-19, Line 419-424) and we strongly recommend interested researchers to fill this gap.

**#25** Since the individual categories being compared here are not exclusive (i.e., a person who experienced x may have experienced y as well), These persons can be completely overlapping. Was this considered in the calculation? Especially when looking at table 3, it shows that nearly half of the persons who did not experience the specific traumatic events also were diagnosed with PTSD.

**Author response:** Thank you very much for raising this issue. Its known that, in war and conflict situation exposure to a multiple traumatic event is potentially high, due to this we assesse a cumulative trauma (frequency of exposure to different traumatic events) and described in methods and material section (page 8, Line 211-214) and its considered in the calculation. We can notice from Table 3 that most of participants are exposed for traumatic events and since exposures are not exclusive, some might expose for a specific event but doesn’t have PTSD but he/she could be exposed for other event to develop PTSD. At the same time, participants could have exposed for other event but didn’t develop the illness.

**#26** In general, it would be great to include more clinical implication.

**Author response:** We appreciate your concern. The widespread implication of PTSD was addressed in the introduction part (page 3, Line 77-81) and the clinical implication in association with anxiety and depressive disorders was addressed in discussion section.

**#27** Line 274-297: It does not have to be so detailed when it comes to the classification of the prevalence rate. I think it would be sufficient to state 1-2 sources with similar background and state that there are also other rates with a similar population but that different reasons have an influence on the rates.

**Author response:** Thank you very much for your constructive comment. We have amended the stating on prevalence rate based on your recommendation.

**#28** Line 308: I am not sure if the authors want to refer to acute stress as a response to destruction/looting or to PTSD.

**Author response:** Thank you very much for pointing out this issue. Individuals with a destruction/looting of personal property creates psychological distress by itself and a significant or difficulty to replace worse the symptoms. A recurrent rehearsal and rumination of the traumatic event becomes their daily experience and some individuals deal effectively but others become impaired and exposed to other mental illnesses including acute stress disorder. In long term untreated cases leads to be post-traumatic stress disorder.

**#29** Line312-313: did the authors ask about the examples of the intrusive thoughts or are these assumptions they made due to the experience of traumatic events?

**Author response:** Thanks for your concern. During the data collection, there were challenging situations specially while facing those who lost their close family members/friends. Most of them talks with disturbed emotion, tearful, sorrow, guilty feelings and anger. We understand the form their words, emotion, facial expression and thoughts that most of them have thought of revenge, anger and self-blame. Additionally, we assume in such un-natural death of family members/friend negative intrusive thought and feeling of revenge might be there, so with both their reporting and our assumption intrusive thoughts may be experienced.

**#30** A source is needed for the stated fact.

**Author response:** Thanks for your critical view. we put a reference for the stated fact.

**#31** Line 361-363: There are other limitations to this study, e.g. that PTSD

**Author response:** Thank you very much for your comment. Based on your comment we revised and add additional imitations. As shown in (page 18, Line 412-419).

# Section Two (Response to comments raised in the second round)

## **Comments from the editor:**

Dear Academic Editor, Thanks for your constructive comments and suggestions in the first round. Now we will address all of comments and concerns raised in the second round as shown below:

Journal Requirements:

Addressing journal requirements.

**#1** Please ensure consistency in capitalization throughout the manuscript, proper use of tittle case for headings and subheadings, and lower case for regular text.

**Author response:** Thank you for your suggestion, to adhere PLOS ONE’s requirements. We have reviewed frequently make correction to ensure the manuscript uses a consistent capitalization, tittle case for heading and subheadings and lower case for regular text throughout the manuscript.

**#2** Please review your reference list to ensure that it is complete and correct.

**Author response:** Thank you for your comment and we checked our reference list repeatedly for its correctness and completeness but we couldn’t found incomplete or retracted references.

**#3** Please be clear about your cumulative measure of cumulative trauma (exposure to the number of different traumatic events). This score should be defined in the method section and also the rational for the categories. Do you find a correlation when using the continuous cumulative trauma score and severity of PTSD (Dose-response relationship)?

**Author response:** Thank you very much for your critical comments. Cumulative trauma was added after a careful revision from different studies. At the beginning, we believe and found evidence of the dose-response association of multiple exposure to traumatic event with PTSD from various studies and decided to assess it in our study by taking the range of categorization from familiar studies conducted in Ethiopia. During analysis stage we checked its correlation in bivariate logistic regression and found a (p-value of 0.877) and rejected from further analysis. Additionally, score of cumulative trauma was defined with its rational in the method section (page 8, Line 211-214) and the result was highlighted in trauma exposure section (page 10, Line 266-268).

**#4** It is quite surprising that there is no cumulative exposure effect in your sample as it has been reported in the previous studies from war affected population. This result should be discussed thoughtfully.

**Author response:** Thank you for your concern. The effect of cumulative trauma (exposure to different traumatic events) with PTSD is known and supported in different studies[6, 7]. We also expect of this, but while checking its association in bivariate regression model, we found (p-value of 0.877) and it was removed from further analysis. In addition to cumulative trauma factors like sex, age group, marital status, educational status, having children, occupation, current/ever substance use, lack of food and water and unlawful imprisonment are also failed to associate. Due to this we discuss on factors significantly associated with the outcome of interest.

**#5** Please clarify the” way of exposure”. Is this type of exposure related to the worst event?

**Author response:** Thank you very much for raising, “way of exposure” is component of criteria “A” on DSM-5 diagnostic criteria[8]. It is the modality through which individuals get exposed to the event. Way of exposure can happen in four means, some individuals directly experience the event on themselves (e.g. Physical trauma/ Serious injury), others might witness the event in person (e.g. Witnessing the murder), by learning that the event occurred to a close family/friend was the other means of exposure and few was exposed as part of their job like police officers, nurses and fire responders. Multiple studies support the direct experience of the event increases the chance of having PTSD as compared with other means of exposure. We assesse” way of exposure” both as part of diagnostic criteria and assessing its correlation with PTSD. During the interview, we assesse individuals for exposure by using Harvard trauma questioner (HTQ) and while encountered with individuals exposed for multiple events we recommend to think of the worst one event then we proceed with the questions.

**#6** Consider another studies about the importance of sexual violence in North East Ethiopia**.** (Tenaw, L. A., Aragie, M. W., Ayele, A. D., Kokeb, T., & Yimer, N. B. (2022). Medical and psychological consequences of rape among survivors during armed conflicts in northeast Ethiopia. PLoS one, 17(12), e0278859.)

**Author response:** Thank you very much for your constrictive comment. We strongly believe the significance of such studies and we mention this as a limitation of our study and we encourage such focused studies to conduct through different methodologies.

**#7** Please use references for the fact of social desirability and underreporting of sexual violence. It may be useful to encourage future studies to assess exposure to sexual violence.

**Author response:** Thank you for concern, social desirability and underreporting of sexual violence items was experienced during our pretest study before the actual data collection, so to encourage researchers we put it as a limitation of our study and we strongly recommend other studies to include rape and sexual violence as part of traumatic events. Since such questions are sensitive and private we recommend to employ qualitative study design and sampling procedures e.g. Snowball sampling techniques.

**#8** In table 3: Clarify why you have put some numbers in bold.

**Author response:** Thank you very much for your critical view, we don’t have any intention rather than indicating variables that significantly associated with the outcome. To adhere with the PLOS ONE’s requirements we un bold those numbers.

**#9** Comments on proper use of upper and lower cases in different sections of the manuscript.

**Author response:** Thank you for your concern. We have made a repetitive review and as much as possible we take amendments on proper use of upper and lower cases throughout the manuscript.

## Reviewers’ comments to the Author

**Reviewer # 1:**

Dear Reviewer #1, Thank you for your constructive comments and suggestions on our manuscript.

**#1** I recommend to the authors to send a point by point response for each questions that has been raised in the previous round.

**Author response:** Dear Reviewer # 1**,** Thank you very much for your comment. In this round, we have incorporated all raised comments in the previous rounds and we gave a point by point response for each question as shown above in section one.

**Reviewer # 2:**

Dear Reviewer #2, Thanks for your constructive comments and suggestions on our manuscript. We have incorporated all concerns and responded for your comments here below:

**#1** In the Introduction section, you only used some parts of the explanation, but I find it important to include the whole explanation as you send it.

**Author response:** Thank you for constructive comment. Based on your request, we make correction to show the whole explanation on the rationale behind conducting this study in Woldia town as shown (page 5, Line 133-136).

**#2** Thank you for your explanation regarding the binary outcome of PTSD. Nevertheless, I find it important to have an idea about the severity of PTSD (knowing the mean is near the cut-off or way above) and it would be great if you can include the M and SD additionally to the binary outcome.

**Author response:** Thank you for your acknowledgment. Up on your request, the Mean was 35.38 and standard deviation was 12.99. The standardized cut-off point of (PCL-5) tool was ≥33 and we can notice that the mean is near the cut-off. The other important thing is PCL-5 tool has only a standardized cut-point but no severity scale as mild, moderate or severe. So, other than putting the M and SD it is difficult to explain its association with the severity scale.

**#3** Could you additionally include that you refer to the DSM-5 PTSD criteria? That would be helpful to know it right away without looking at the reference. Thank you.

**Author response:** Thank you very much for your comment. In the introduction, we use the standardized definition of PTSD sourced from DSM-5 and cited appropriately. Based on your comment, we mention our reference (DSM-5) for defining PTSD, as shown (page 3, Line 62).

**#4** Thank you for including global figures and other surveys. This improved the introduction. For a more focused narrative, I would suggest deleting the studies from Ukraine, Syria and Lebanon because you refer to so many other countries in Africa that this already makes your point.

**Author response:** Thank you very much for your suggestion. To show the global burden, we only highlight recent studies conducted from different corners of the world and we gave a strong emphasis on studies conducted in Africa.

**#5** In addition, I would like to encourage you to add more details in the section where you address contributing factors to the occurrence of PTSD (ll 121-128) if they are from studies conducted in Africa

**Author response:** We thank you for your important view. most of studies cited in the contributing factors are studies conducted in Africa including Ethiopia as shown (Page 5, Line 122-129).

**#6** Thank you for explaining the criteria regarding “severely sick” or “unable to communicate”. It would be helpful if you include this explanation also in the manuscript.

**Author response:** We really thank you and appreciate. Based on your suggestion, we also include the explanation in the manuscript as shown (Page 6, Line 164-167).

\#**7** Comment #11 and #18 Thank you for the clarification. Nevertheless, it would be great to know why the p-value is <0.2 and <0.25. I do not fully understand the decision behind the p-values.

**Author response:** We are grateful for your comment. Here, we want to clarify that p-values are decided based on the numbers of factors associated with the outcome of interest. Exact number of factors are not clearly stated[9], but if factors are adequate enough you can take variables with p-values <0.2. In our study, we found eight variables (Destruction/looting of property, murder/ serious injury of a family member/friend, a witness of the murder of family member/ friend, unlawful imprisonment, experience traumatic event on themselves and having a poor and moderate level of social support, depression and anxiety) and if we take <0.25 the factors might rise and we lose our focus while addressing to all of them. So, we take variables with p-value <0.2 and focus on those eight factors.

Additional new comments:

\#**8** Table 2: Please add the frequencies for male and female in the columns. At the moment you report the sample size for male and female but due to different sizes, it is hard to compare both columns. You already have the frequency for the total sample and it would be helpful to also have it for male/female.

**Author response:** Thanks for pointing out again. Dear Academic Editor/Reviewer #2, this question was raised in the previous round and addressed properly, you can check it in section one #18 and as shown in (Page 10-11, Table 2), we have described the frequency of exposure to traumatic events based on sex (male/female) in the columns.

\#**9** Table 3: Please include the abbreviation AOR and CI in the notes below the table.

**Author response:** Thank you for your recommendation. We have included the all abbreviations used in the manuscript in the List of Abbreviation section, as shown in (page 19, Line 438-441).

# Additional Editor Comments from the last review:

Dear Academic Editor, Thanks for your prompt and constructive comments and suggestions on our manuscript. We have accepted suggestions and respond to all raised concerns here below:

\#**1** Thanks you for including more information on the cumulative trauma score that you used in the study. However, this sentence needs revision in order to improve language.

**Author response:** Thanks again for your important suggestion. We have seen the revised sentences and found its better ways of explanation with improved language and we amended the sentence based on your suggestion.

**#2** Please revise this once more if this score pertains to the frequency of exposure to the same traumatic event or to the exposure to different types of traumatic events.

**Author response:** We thank you for your concern. We want to make clear that cumulative trauma pertains the exposure to different types of traumatic events. Based on this, we make corrections in method section (page 8, Line 211-214) and result section (page 10-11, Table 2).

**#3** Please discuss the deviance from a lot of findings form the literature in your discussion.

**Author response:** Thanks for your concern. We agree that cumulative trauma was not significantly associated due to this eliminated from further analysis and at the same time, eight important factors were significantly associated with the outcome and our discussion gives a total focus on those factors. In another side, adding cumulative trauma in multivariate analysis was not a right procedure that, it affects our control over possible confounders, causes important factors fails to associate and results an overall statistical bias.

**#4** Your sentence has some minor grammatical issues:

**Author response:** We thank you for suggestion. We read the suggested sentence with correction and we decide to take the revised one.

**#5** For reviewer 2, I have found that you have deleted some parts of the comment. Please go back to the comments and present complete reviewer comments.

**Author response:** Dear Academic Editor and Reviewer #2, We have addressed most of the questions raised by the reviewer #2 but some statements are tricky, that it contains two and three questions inside one statement and after responding to the first we jump to the next item and due to this we missed some questions unintentionally. We sincere apology for missing and we found those questions. Item #20, #21 and #26 in section one above are the added comments and addressed properly.

**#6** Response to comment 1: Please make clear how you address this issue in your paper. There seemed to be no changes made.

**Author response:** Thanks for raising. We have addressed for the question but we failed to incorporate it in the manuscript. Based on your request, we include the addressed point in the introduction section as shown (page 5, Line 133-136).

\#**7** The reviewer has kindly asked you to present M and SD of the PTSD severity score.

**Author response:** Thank you for your concern. Up on your request, the Mean was 35.38 and standard deviation was 12.99. The standardized cut-off point of (PCL-5)[5] tool was ≥33 and we can notice that the mean is near the cut-off. The other important thing is PCL-5 tool has only a standardized cut-point (binary outcome) but no severity scale like mild, moderate or severe. So, other than putting the M and SD it is difficult to explain its association with the severity scale.

\#**8** # 11 and # 18 please make your decision clear regarding the p-values.

**Author response:** We are grateful for your comment. This comment was addressed above and Here; we want to clarify that p-values are decided based on the numbers of factors associated with the outcome of interest. Exact number of factors are not clearly stated, but if you believe factors are adequate enough you can take variables with p-values <0.2. In our study, we found eight variables (Destruction/looting of property, murder/ serious injury of a family member/friend, a witness of the murder of family member/ friend, unlawful imprisonment, experience traumatic event on themselves and having a poor and moderate level of social support, depression and anxiety) and if we take <0.25 the numbers might rise and we lose our focus while addressing to all of them. So, we decide to take p-value <0.2 and focus on those eight factors.

\#**9** Table 2. Please report the frequencies for each sex as requested by the reviewer despite presenting the general frequency. I would recommend to present the frequency for each sex in brackets in Table 2. Please respect the comment regarding Table 2.

**Author response:** Thanks for rising this. Dear Academic Editor and Reviewer #2, We respect every raised questions during the whole process, but this question was repetitively raised in the previous round and addressed properly, you can check it in section one item #18 and section two (Additional new comments) item #8. And as shown in the “Revised Manuscript with Track Changes” file (Page 10-11, Table 2), we have described the frequency of exposure to traumatic events for each sex (male/female) in the columns.

1.	Ali D, Azale T, Wondie M, Tadesse JJPr, management b. About six in ten survivors of the november 2020 Maikadra massacre suffer from posttraumatic stress disorder, northwest Ethiopia. 2022;15:251.

2.	Manea L, Gilbody S, McMillan DJC. Optimal cut-off score for diagnosing depression with the Patient Health Questionnaire (PHQ-9): a meta-analysis. 2012;184(3):E191-E6.

3.	Spitzer RL, Kroenke K, Williams JB, Löwe BJAoim. A brief measure for assessing generalized anxiety disorder: the GAD-7. 2006;166(10):1092-7.

4.	Kocalevent R-D, Berg L, Beutel ME, Hinz A, Zenger M, Härter M, et al. Social support in the general population: standardization of the Oslo social support scale (OSSS-3). 2018;6(1):1-8.

5.	Weathers FW, Litz BT, Keane TM, Palmieri PA, Marx BP, Schnurr PPJSaftNCfPawpvg. The ptsd checklist for dsm-5 (pcl-5). 2013;10(4):206.

6.	Madoro D, Kerebih H, Habtamu YJND, Treatment. Post-traumatic stress disorder and associated factors among internally displaced people in south ethiopia: a cross-sectional study. 2020;16:2317.

7.	Seino K, Takano T, Mashal T, Hemat S, Nakamura KJH, outcomes qol. Prevalence of and factors influencing posttraumatic stress disorder among mothers of children under five in Kabul, Afghanistan, after decades of armed conflicts. 2008;6(1):1-5.

8.	American Psychiatric Association D, Association AP. Diagnostic and statistical manual of mental disorders: DSM-5: American psychiatric association Washington, DC; 2013.

9.	Kirkwood BR, Sterne JA. Essential medical statistics: John Wiley & Sons; 2010.
